# **β**-Caryophyllene, a Compound Isolated from the Biblical Balm of Gilead (*Commiphora gileadensis*), Is a Selective Apoptosis Inducer for Tumor Cell Lines

**DOI:** 10.1155/2012/872394

**Published:** 2012-04-10

**Authors:** Eitan Amiel, Rivka Ofir, Nativ Dudai, Elaine Soloway, Tatiana Rabinsky, Shimon Rachmilevitch

**Affiliations:** ^1^French Associates Institute for Agriculture and Biotechnology of Drylands, Ben Gurion University of the Negev, Sede-Boqer Campus, Midreshet Ben Gurion 84990, Israel; ^2^Dead Sea and Arava Science Center, the Dead Sea 86910, Israel; ^3^Center for Sustainable Agriculture, Arava Institute for Environmental Studies, D.N. Hevel Eilot 88840, Israel; ^4^The Unit of Medicinal and Aromatic Plants, Newe Ya'ar Research Center, Ramat Ishai 30095, Israel; ^5^Department of Microbiology & Immunology, Ben-Gurion University of the Negev, Beer-Sheva 84105, Israel

## Abstract

The biblical balm of Gilead (*Commiphora gileadensis*) was investigated in this study for anticancerous activity against tumor cell lines. The results obtained from ethanol-based extracts and from essential oils indicated that **β**-caryophyllene (trans-(1R,9S)-8-methylene-4,11,11-trimethylbicyclo[7.2.0]undec-4-ene) is a key component in essential oils extracted from the balm of Gilead. **β**-Caryophyllene can be found in spice blends, citrus flavors, soaps, detergents, creams, and lotions, as well as in a variety of food and beverage products, and it is known for its anti-inflammatory, local anaesthetic, and antifungal properties. It is also a potent cytotoxic compound over a wide range of cell lines. In the current paper, we found that *Commiphora gileadensis* stem extracts and essential oil have an antiproliferative proapoptotic effect against tumor cells and not against normal cells. **β**-caryophyllene caused a potent induction of apoptosis accompanied by DNA ladder and caspase-3 catalytic activity in tumor cell lines. In summary, we showed that *C. gileadensis* stems contain an apoptosis inducer that acts, in a selective manner, against tumor cell lines and not against normal cells.

## 1. Introduction

For a normal living cell to replicate accurately, it must go through several processes: precisely reproduce its DNA, produce sufficient cellular organelles, membranes, and so forth, to enable the survival of the daughter cells, and divide its DNA, cytoplasm, and organelles equally to form two functioning daughter cells [1–3]. These processes are part of a well-controlled equilibrium which exists between cell proliferation and cell death, two stages of the cell cycle that have become appreciated targets for intervention against cancer as the cancerous process results from an imbalance between the two [1]. This imbalance is linked with changes in the cell's response to signals from within. These signals include: self-sufficiency in growth signals, insensitivity to growth-inhibitory (antigrowth) signals and limitless replicative potential (over proliferation), sustained angiogenesis, tissue invasion, metastasis, and evasion of apoptosis. Each of these physiologic changes occurs during tumor development [1, 3, 4].

Apoptosis, the programmed cell death, comprises two main phases: a commitment to cell death followed by an execution phase [5]. The apoptotic process is a cascade reaction, carried out and regulated by specialized cellular machinery, a family of cysteine proteases called caspases. The appearance of these caspases seems to be the biochemical event that defines a cellular response as apoptosis more than any other [6]. The caspase family is divided into two: the initiator caspases (caspase-8 and caspase-9) and the executioner caspases (caspase-3, -6, and -7) [7]. An apoptotic death stimulus (stimuli is plural) activates the initiator caspases which, in turn, activate the executioner caspases. The active executioners promote apoptosis by cleaving cellular substrates, which induces the morphological and biochemical features of apoptosis [8–10].

In modern cancer research, the isolation of active compounds from natural products for drug use has reached an all-time high in modern westernized medicine, resulting in most of the existing anticancer drugs [11, 12]. These drugs are aimed specifically at various components of the intracellular signal transduction pathways controlling cell cycle, programmed cell death (apoptosis), or angiogenesis [9]. These naturally derived products are offering the world of medicine a great opportunity to evaluate new chemical classes of anticancer agents, as well as novel mechanisms of action [13].

In this study, we describe a new apoptosis-inducing agent, *β*-caryophyllene (*trans*-(1R,9S)-8-methylene-4,11,11-trimethylbicyclo[7.2.0]undec-4-ene) which was found to be a major component in the essential oil derived from *C. gileadensis*. *β*-caryophyllene, a compound found in spice blends, citrus flavors, soaps, detergents, creams and lotions, and also in a variety of food and beverage products, [14, 15] is known for its anti-inflammatory, local anaesthetic, and antifungal properties [16–18]. In experiments conducted on tumor cell lines, *β*-caryophyllene has been reported to have a potent cytotoxic activity over a wide range of cell lines [19, 20].

**Figure 1:**
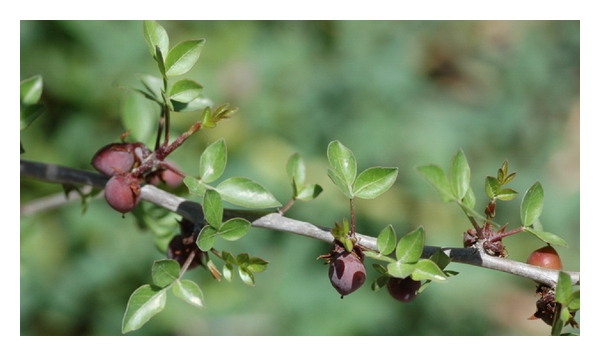
*C. gileadensis* leaves and fruits. Photo taken in Ein Gedi's botanical gardens. Photos taken by Shelef Oren.

**Figure 2:**
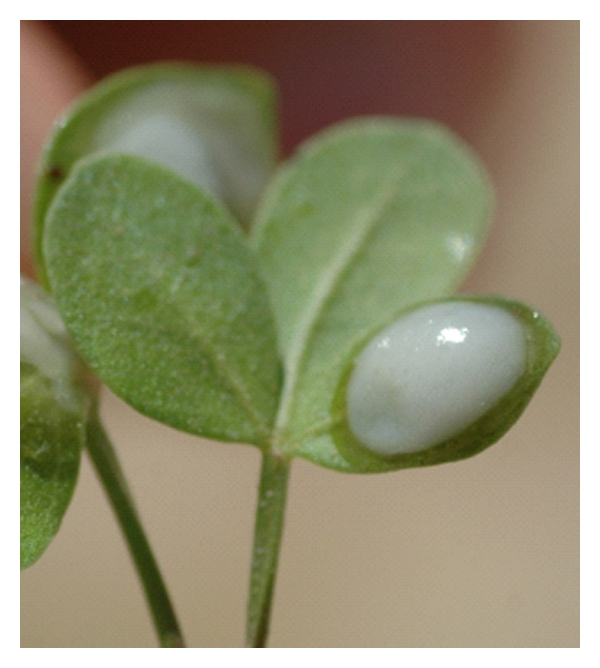
A drop of *C. gileadensis* resin on a* C. gileadensis* leaf. Photo taken in Ein Gedi's botanical gardens. Photos taken by Shelef Oren.

*Commiphora gileadensis *(L.) (*Syn C. opobalsamum*,* Amyris gileadensis*,* Amyris opobalsamum*,* Balsamodendron opobalsamum *[21]) is a member of the highly studied and commercially used resinous plant family Burseraceae, comprising, among others, the biblical frankincense and myrrh [22]. It is native to southwest Arabia and Somaliland, where it grows in a thorn-bush formation under arid tropical conditions, often accompanied by shrubs and trees such as *Balanites aegyptiaca* and species of *Maerua*, *Ziziphus* [23]. It is a thornless shrub (1.5 m high) or small tree (5 m high) with a reddish or grayish bark which grows in hot deserts and semideserts. Its white flowers create small clusters and produce ovoid or ellipsoid, smooth, glabrous fruits which contain a fragrant yellow seed that brightens in color as the seed matures and dries [23–25] (Figures 1 and 2). After receiving a specimen sheet from his student Peter Forsskål in Yemen, Linnaeus gave it the name *Commiphora gileadensis*, in connection with the Bible's “balm of Gilead” (Genesis 37 : 25, Jeremiah 46 : 11, and Jeremiah 8 : 22). The specimen was sent with a letter dated June 3rd 1763, in which Forsskål wrote about his discovery of the “balm of Gilead” [21, 25, 26]. Ancient writers, such as Josephus Flavius, Pliny the elder, Pedanius Dioscorides, and Gaius Tacitus, highly praised it (the balm (balsam) tree of Judea) for its use as holy oil and in perfumes, but also considered it as a cure for many diseases [23, 27]. The shoots of *C. gileadensis* are used in folk medicine to treat various illnesses [28–33]. However, the history and elaborate folk medicinal value of the species has not yet been put to the test and remains generally overlooked by modern science.

In this manuscript, we describe the antiproliferative and proapoptotic effect of the *C. gileadensis*-derived essential oil and its derivative compound *β*-caryophyllene. 

## 2. Materials and Methods

### 2.1. Plant Material

*Commiphora gileadensis* cuttings were obtained from the Dead Sea Ein Gedi Botanical Garden located in kibbutz Ein Gedi, the Dead Sea, Israel (31° 27′N, 35° 23′E) (plants originated from the Chelsea Physic Garden, 66 Royal Hospital Road, Chelsea, London, UK). In Ein Gedi's botanical gardens, the plants were grown in the field in sandy soil and drip irrigated with tap water (drinking water originating from Ein Gedi's spring water). Each plant received 10 liters every four days. Between November and March, the plants were watered every five days. The plants were identified as *Commiphora gileadensis* (L.) (Burseraceae), by Dr. Shimon Rachmilevitch from the Department of Biotechnology and Agriculture in Arid Land in Ben Gurion University.

### 2.2. Extraction

*Commiphora gileadensis *stem extracts were prepared as follows.

1. Ethanolic stem extracts: stems were dried at 40°C for three days and stem powder was suspended in tubes with ethanol 96% (EtOH-FRUTAROM) at a ratio of 200 *μ*g/mL which were incubated overnight at room temperature (25°C), followed by centrifugation (13,000 revolutions per minute (rpm)). The supernatant (the extract) was moved to another tube and kept at −20°C until used.
2. MTBE extraction for volatiles analysis: plant stems were extracted with methyl-*tert*-butyl ether (MTBE), containing 10 mg/mL isobutylbenzene as an internal standard, for 24 h with gentle shaking at room temperature [34].

### 2.3. Chemical Compounds

- *β*-Caryophyllene: (−)-*trans*-caryophyllene, syn. *β*-Caryophyllene, *trans*-(1R,9S)-8-methylene-4,11,11-trimethylbicyclo[7.2.0]undec-4-ene (C_15_H_24_), was purchased from Sigma-Aldrich, Inc. 204.35 g/mol, ≥98.5% pure, catalog number 22075.
- Citral: syn. 3,7-dimethyl-2,6-octadienal (C_10_H_16_O), 40 : 60% of geranial and neral mixture was used in this research as a positive control [35]. Purchased from Sigma-Aldrich, Inc (Fluka). 152.24 g/mol, ≥95% pure, catalog number 27450.
- Staurosporine (STS): syn. antibiotic AM-2282 (C_28_H_26_N_4_O_3_), from *Streptomyces *sp., was used in this research as a positive control. Purchased from Sigma-Aldrich, Inc. 466.53 g/mol, ≥95% pure, catalog number S4400.

### 2.4. Cell Culture

The following cell lines were used in this study.

  BS-24-1: mouse lymphoma cell line (T cells, [36]).
  MoFir: Epstein-Barr virus-transformed human B lymphocytes generated in the laboratory by transformation of a human B cell from an anonymous donor (see below).
  FB: normal human skin fibroblasts (BJ cells, [36]).

BS-24-1, and MoFir were grown in a Roswell Park Memorial Institute medium (RPMI, Biological Industries Beit Haemek) supplemented with 2 mM L^−1^ glutamine (Biological Industries Beit Haemek), 10% fetal bovine serum (FBS, Biological Industries Beit Haemek), 100 U/mL penicillin, and 100 mg/mL streptomycin (Biological Industries Beit Haemek). FBs was grown in a Dulbecco/Vogt modified Eagle's minimal essential medium (DMEM-Biological Industries Beit Haemek) supplemented with 2 mM L^−1^ glutamine, 20% FBS, 100 U/mL penicillin and 100 mg/mL streptomycin. All the cells were maintained at 37°C in a water-saturated atmosphere of 5% CO_2_.

### 2.5. EBV Transformation

To establish the MoFir cells, B cells from whole blood were separated by Ficoll-Hypaque density gradient centrifugation. The cells were infected with the B95-8 strain of the Epstein-Barr virus. The RPMI medium (Biological Industries Beit Haemek) was used for cell culture. The EBV B lymphocyte cell line was maintained in RPMI, supplemented with 10% FBS, 100 U/mL penicillin, and 100 mg/mL streptomycin. The culture was maintained at 37°C in 5% CO_2_ atmosphere. The medium was changed every three days.

### 2.6. GC-MS Analysis

One *μ*L from the extracted samples was analyzed in a computerized GC-MS (GC-6890N) equipped with a mass selective (MS)—5973 network (electron ionization 70 eV) detector of Agilent Technologies (CA, USA). A capillary column, Rtx-5Sil MS (Restek Corporation, State College, PA, USA) (30 m × 0.25 mm) i.d × 0.25 *μ*m silica, was installed into the GC-MS. The carrier gas, helium (He), was in a mode of constant flow of 1 mL/min. The extraction samples were introduced into the column in a “splitless” mode while the oil and SPME samples were introduced in a “split” mode ratio of 1 : 50. Temperatures were set as follows: the injector's temperature was 250°C, and both the transfer line and detector's temperature were 280°C. The columns' temperature gradient was set at 50°C for 1 minute, with additions of 5°C per minute up to 260°C, and then 260°C for ten minutes.

Component recognition was based on a comparison of the retention time index (RI) of the components to commercial standards and by comparison of the samples' mass spectrum with GC-MS libraries: Adams 2001, NIST 98, and QuadLib 1607.

### 2.7. Essential Oil Preparation

Essential oils of *Commiphora gileadensis* (L.) (Burseraceae) (Gilead oil) were prepared as described [34].

Three dilutions were examined in the experiment: 1 : 20000, 10000, and 5000. Stock solutions of Gilead oil dilutions were prepared by adding 1 *μ*L to 20, 10, and 5 *μ*L ethanol, respectively. 1 *μ*L from the stock solution was added into the 1000 *μ*L cell growth medium.

### 2.8. Results Presentation

The results presented in this study are from a representative experiment. A minimum of five experiments were carried out with each compound, and the samples were analyzed in triplicates.

Cell death was assessed by the incubation of cells with Tetrazolium bromide salt MTT. The production of formazan was monitored using an ELISA reader at a wavelength of 450 nm. Cytotoxicity in the compound-treated culture was expressed as follows:

(1) $$\begin{array}{c} \%\;\text{death}=100\times \left(\frac{\text{absorbance}\;\text{of}\;\text{compound-treated}\;\text{cell}}{\text{absorbance}\;\text{of}\;\text{ethanol-treated}\;\text{cells}}\right). \end{array}$$

Apoptosis was induced by incubating the cell lines with the compounds in their normal serum-supplemented growth medium.

The caspase-3 cellular activity assay was carried out according to the manufacturer's instructions. For measuring the specific inhibition by Ac-DEVD-CHO, cell extracts were preincubated with the inhibitor (0.05 *μ*M) for 10 minutes before the addition of the substrate. The DNA ladder analysis was performed as described before [37].

## 3. Results

**Figure 3:**
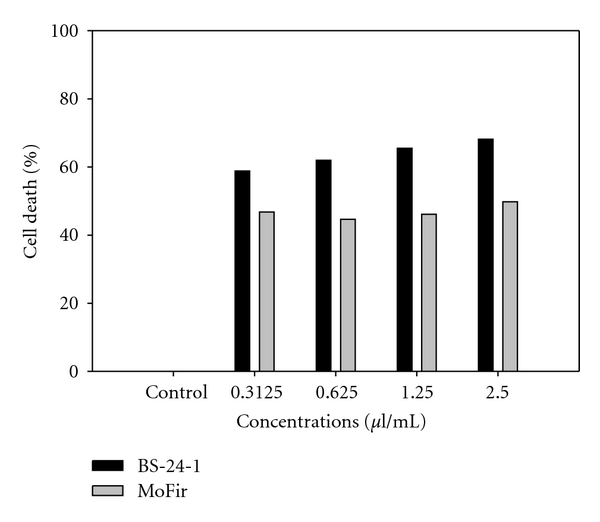
Growth inhibitory effect of ethanol-based *Commiphora gileadensis* stem extract on two tumor cell lines. Extract concentrations shown on *x*-axis are in *μ*L/mL. The cells were plated at a concentration of 500,000 cells/mL and incubated with and without stem extract for 17 h. Control cells were treated with ethanol (0.05%) alone.

We first investigated whether *C. gileadensis* extracts had antiproliferative effects against tumor cell lines using the MTT assay. We evaluated its effect on mouse (BS-24-1) and human (MoFir) cell lines using ethanol- (EtOH-) based stem extracts. As shown in Figure 3, following 24 h incubation of cells with extracts, cell survival was 30 and 50% for BS-24-1 cells and MoFir cells at 0.5 *μ*L/well, respectively. IC_50_ was 0.3125 and 2.5 *μ*L/mL, respectively.

**Figure 4:**
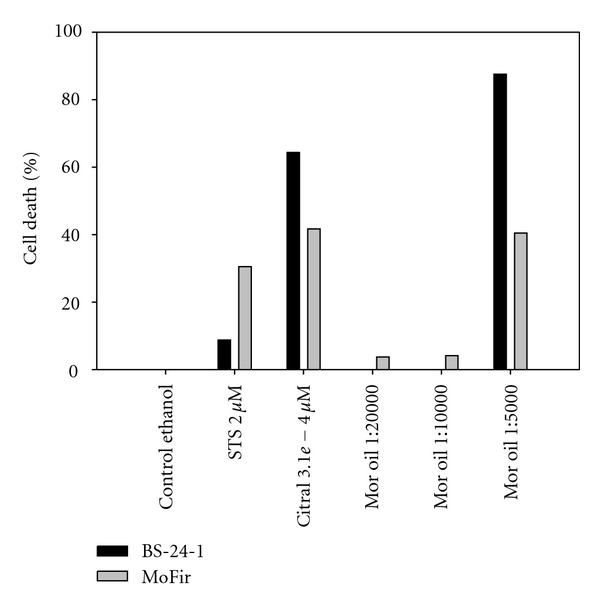
Growth inhibitory effect of *C. gileadensis* essential oil on two tumor cell lines. The cells were plated at a concentration of 500,000 cells/mL and incubated with citral (3.1*e* − 4 *μ*M), STS (2 *μ*M) and three concentrations of essential oil from *C. gileadensis *(named here gilead oil) for 2 h. Control cells were treated with ethanol (0.05%) alone.

**Figure 5:**
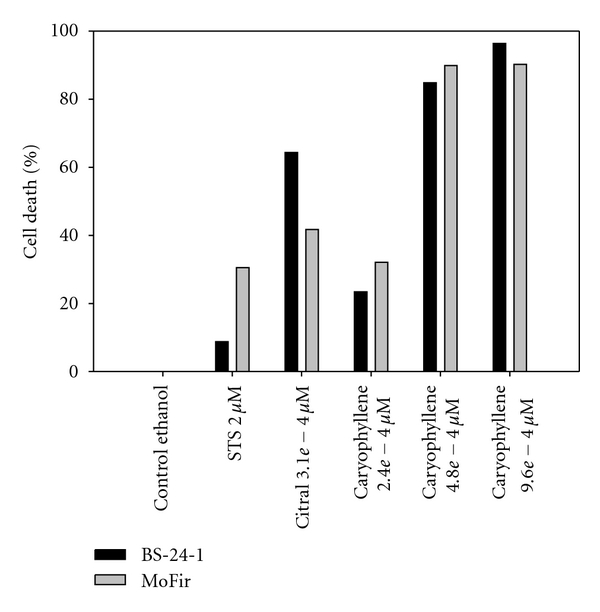
Growth inhibitory effect of *β*-caryophyllene on two tumor cell lines. The cells were plated at a concentration of 500,000 cells/mL and incubated with citral (3.1*e* − 4 *μ*M), STS (2 *μ*M) and three *β*-caryophyllene concentrations (2.4*e* − 4, 4.8*e* − 4, and 9.6*e* − 4 *μ*M) for 2 h. Control cells were treated with ethanol (0.05%) alone.

Based on the latter results, we assessed the effect of *C. gileadensis *essential oil on tumor cell lines, comparing it to two reference compounds: staurosporine (STS) and citral [35] (Figure 4). Proliferative inhibition of Gilead oil incubated with tumor cell lines for 2 h showed concentration dependency (Figure 4), and Gilead oil concentration of 1 : 5000 killed 87% of BS-24-1 cells and 40% of MoFir cells, suggesting that one or more of the components in the essential oil contributed to the tumor cell lines killing effect. We next separated and identified the Gilead oil volatiles components using GC-MS. The compound list received from the fractionation (Table 1) was composed mainly of terpens from which we decided to focus on what was most likely to be the active compound, *β*-caryophyllene, known for its cytotoxicity, anti-inflammatory, and antifungal activity [16–20, 38]. To examine the effect of *β*-caryophyllene, both tumor cell lines were incubated for 2 h with *β*-caryophyllene while STS and Citral served as references (Figure 5). *β*-Caryophyllene showed a concentration dependency of proliferation inhibition and induced 85–90% cell killing in both cell lines at concentrations of 4.8*e* − 4 *μ*M. These results suggest that *β*-caryophyllene is the active compound within the oil extract.

**Figure 6:**
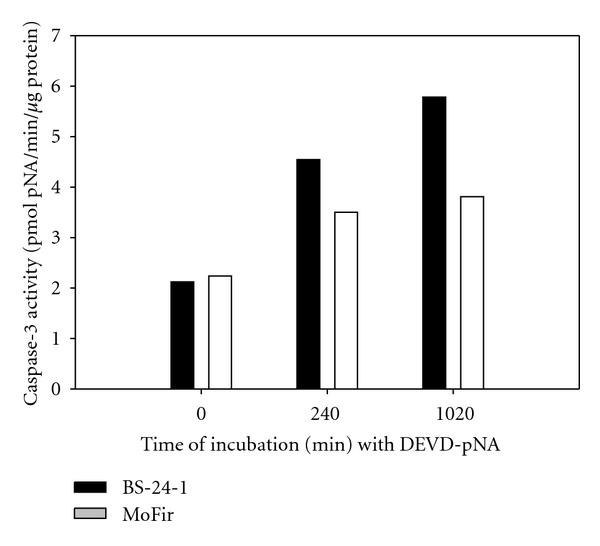
Apoptogenic effects of *β*-caryophyllene on two tumor cell lines. Enzymatic activity of different cell lines incubated with *β*-caryophyllene (2.4*e* − 4 *μ*M) for 2 h. *x*-axis represents the time of the enzymatic reaction *in vitro* in minutes.

**Figure 7:**
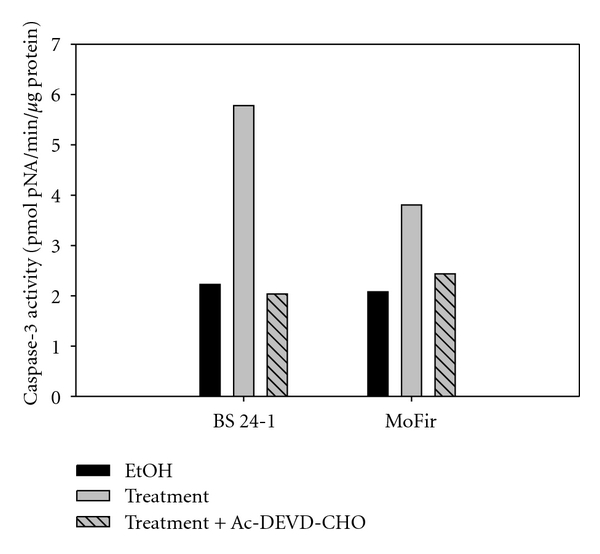
*β*-caryophyllene activate caspase-3 in two tumor cell lines. The enzymatic activity of different cell lines incubated with *β*-caryophyllene (2.4*e* − 4 *μ*M) for 2 hrs was assessed. Measurements were taken following 1020 min of enzymatic reaction *in vitro*. Preincubation with caspase-3 inhibitor (DEVD-CHO) eliminated any development of caspase-3 activity.

**Figure 8:**
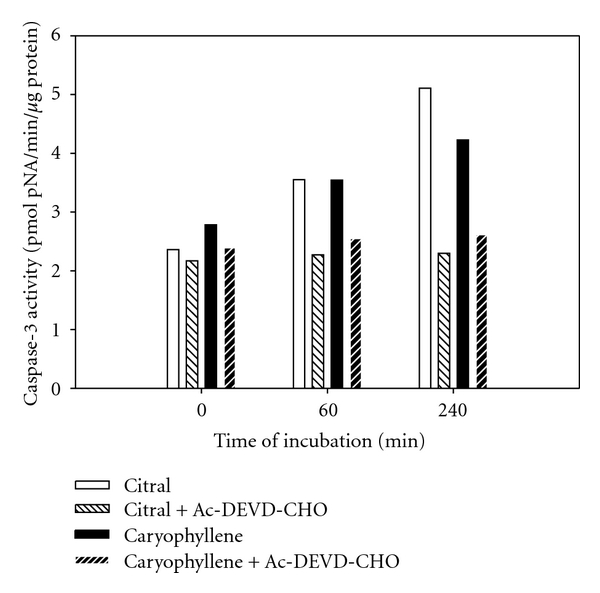
Comparison of the apoptogenic effects of *β*-caryophyllene and citral on BS-24-1 tumor cell lines. Enzymatic activity of BS-24-1 incubated with *β*-caryophyllene (0.33 *μ*L/mL) and citral (0.1 *μ*L/mL) for 2 hrs. *x*-axis represents the time of the enzymatic reaction *in vitro* in minutes. Preincubation with caspase-3 inhibitor (DEVD-CHO) eliminated any development of caspase-3 activity.

Incubation of human (MoFir) and mouse (BS-24-1) tumor cell lines for 2 h with *β*-caryophyllene (2.4*e* − 4 *μ*M) resulted in the activation of the enzymatic activity of caspase-3 (Figure 6). Pretreatment of Mofir and BS-24-1 cells with the specific caspase-3 inhibitor, Ac-DEVD-CHO, inhibited caspase-3 activity, indicating that the active enzyme in the assay in both cell lines is indeed caspase-3 (Figure 7). The property of *β*-caryophyllene as an inducer of caspase-3 enzymatic activity was compared and found similar to the reference compound citral (Figure 8).

**Figure 9:**
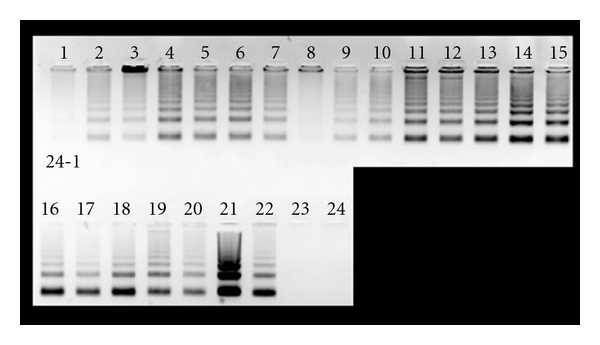
BS-24-1 cells (1,000,000 cells/mL) were incubated with *C. gileadensis* stem and leaf extracts (6 *μ*L/mL) for 17 h. DNA was separated and analyzed on agarose gel. Lanes 1–7 and 11: H_2_O-based stem extract. Lanes 8–10: H_2_O-based leaf extract. Lanes 12–18 and 22, ethanol-based stem extract. Lanes 19–21: ethanol-based leaf extract. Lanes 23 and 24: control cells treated with ethanol (0.05%) and water.

**Figure 10:**
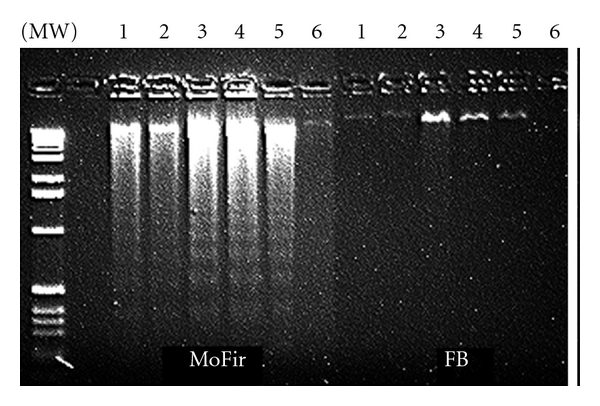
MoFir and FB cells were incubated with *C. gileadensis* extract (2.5 *μ*L/mL) for 24 h. DNA was separated and analyzed on agarose gel. Lanes 1 and 2: ethanol-based stem extract. Lane 3–5: ethanol-based leaf extract. Lane 6: untreated cells with ethanol (0.05%) alone.

**Figure 11:**
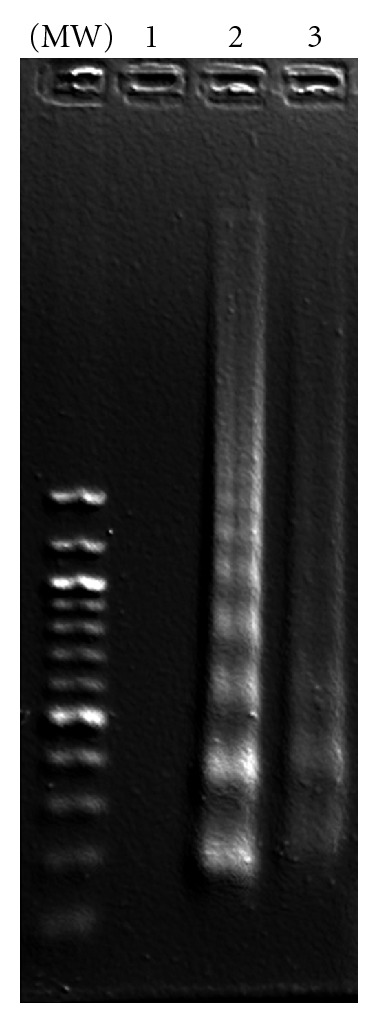
BS-24-1 cells were incubated with *C. gileadensis* essential oil for 24 h. DNA was separated and analyzed on agarose gel. Lane 1: control-cells treated with ethanol (0.05%) alone. Lane 2: cells incubated with 0.625 *μ*L/mL essential oil. Lane 3: cells incubated with 1.25 *μ*L/mL essential oil.

**Figure 12:**
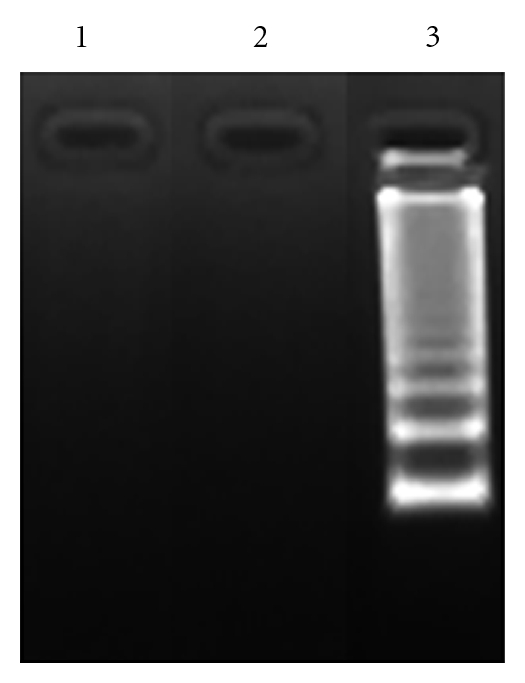
Fragmentation of DNA in the presence of *β*-caryophyllene. BS-24-1 cells were incubated in the presence of *β*-caryophyllene in two combinations of time and concentrations. Lane 1: cells with ethanol (0.05%) alone. Lane 2: cells incubated for 24 h with 2.4 *μ*lM. Lane 3, cells incubated for 2 h with 9.6 *μ*M *β*-caryophyllene.

A biochemical hallmark of apoptosis is the activation of endonucleases, leading to the fragmentation of the genomic DNA, which produces a characteristic ladder when separated on an agarose gel electrophoresis [39]. When *C. gileadensis *stem extracts were incubated with BS-24-1 for 24 h, a DNA ladder was observed (Figure 9). The incubation of *C. gileadensis *stem extracts with normal cells (FB) and MoFir for 24 h produced a DNA ladder only in MoFir (the tumor cell line, Figure 10). 24 h incubation of Gilead oil with BS-24-1 formed a similar pattern (Figure 11) that was repeated following 2 h treatment of BS-24-1 cells with *β*-caryophyllene (Figure 12). These results suggest that the *C. gileadensis *stem extract is an apoptosis inducer that acts in a selective manner against tumor cell lines and not against normal cells. 

## 4. Discussion

*Commiphora gileadensis* stem extracts and essential oil showed antiproliferative proapoptotic effects (exhibited via DNA “ladder” and caspase-3 activation) in tumor cell lines, while there was no apoptosis induction in normal cell lines (FB) (Figure 10). *β*-Caryophyllene, one of the compounds in the essential oil, is responsible for these effects. Our results agree with previous studies [16–20, 38] which indicated that *β*-caryophyllene shows antiproliferative activity against tumor cell lines. To the best of our knowledge, this is the first time that the function of an apoptosis-inducing agent was assigned to *β*-caryophyllene. One of the main problems with compounds used for chemotherapy is that they are nonselective, namely, they kill normal cells as well as cancer cells [40]. Interestingly, the results in the current study indicated that *β*-caryophyllene acts in a selective manner against tumor cell lines and not against normal cells. Since *β*-caryophyllene is a natural product used by humans on a daily basis [14, 15], it may be readily acceptable as a dietary supplement with medicinal values capable of eradicating tumor cells and, as such, may protect against tumor development.

## Figures and Tables

**Table 1:** Composition of the essential oil from *C. gileadensis* leaves and fruits collected in Ein Gedi's botanical garden, on June 2nd, 2009. General chemical profile and the content percentage of individual components are presented.

Components	% content in oil
*α*-Thujene	0.64
*α*-Pinene	7.21
Camphene	0.18
Sabinene	21.11
*β*-Pinene	0.90
*α*-Terpinene	—
Para-Cymene	0.16
Limonene	0.27
*β*-Phellandrene	0.80
(Z)-*β*-Ocimene	0.34
(E)-*β*-Ocimene	—
*γ*-Terpinene	2.75
*cis*-Sabinene hydrate	0.16
Terpinolene	—
*Trans*-Sabinene hydrate	0.20
allo-Ocimene	0.28
Borneol	—
Terpinen-4-ol	1.26
*α*-Terpineol	0.33
*n*-Decanal	—
Bornyl acetate	1.66
Bicycloelemene	2.21
*α*-Ylangene	0.63
*β*-Cubebene	0.10
*β*-Elemene	0.53
(*E*)-Jasmone	0.17
*β*-Caryophyllene	20.12
*β*-Copaene	1.85
6,9-Guaiadiene	0.43
Sesquiterpene hydrocarbone	0.77
Sesquiterpene hydrocarbone	0.69
Sesquiterpene hydrocarbone	1.29
*α*-Humulene	0.59
Dauca-5,8-diene	0.43
Germacrene D	19.62
Bicyclogermacrene	2.91
Sesquiterpene hydrocarbone	1.02
*δ*-Amorphene	1.35
*γ*-Cadinene	2.65
*δ*-Cadinene	0.37
Nerolidol	0.31
Germacrene D-4-ol	0.55

## Abbreviations

BS-24-1: Mouse lymphoma cell line
EBV: Epstein-Barr virus
EtOH: Ethanol
FB: Normal human skin fibroblasts
MoFir: Epstein-Barr virus-transformed human B lymphocytes.
